# Genome Sequences of Both Organelles of the Grapevine Rootstock Cultivar ‘Börner’

**DOI:** 10.1128/MRA.01471-19

**Published:** 2020-04-09

**Authors:** Bianca Frommer, Daniela Holtgräwe, Ludger Hausmann, Prisca Viehöver, Bruno Huettel, Reinhard Töpfer, Bernd Weisshaar

**Affiliations:** a Bielefeld University, Chair of Genetics and Genomics of Plants, Faculty of Biology & Center for Biotechnology (CeBiTec), Bielefeld, Germany; b Julius Kühn Institute, Institute for Grapevine Breeding Geilweilerhof, Siebeldingen, Germany; c Max Planck Genome Centre Cologne, Max Planck Institute for Plant Breeding Research, Cologne, Germany; University of California, Riverside

## Abstract

Genomic long reads of the interspecific grapevine rootstock cultivar ‘Börner’ (Vitis riparia GM183 × Vitis cinerea Arnold) were used to assemble its chloroplast and mitochondrion genome sequences. We annotated 133 chloroplast and 172 mitochondrial genes, including the RNA editing sites. The organelle genomes in ‘Börner’ were maternally inherited from *Vitis riparia*.

## ANNOUNCEMENT

Long reads generated by single-molecule real-time (SMRT) DNA sequencing technology (Pacific Biosciences) are one starting point for high-quality chloroplast (1, 2) and mitochondrion genome sequence assemblies. The cultivated grapevine Vitis vinifera is highly susceptible to pathogens. Resistant cultivars like the interspecific hybrid ‘Börner’ (V. riparia GM183 [mother plant] × V. cinerea Arnold [pollen donor]) are used as rootstocks for growing elite grapevine varieties. We assembled and annotated the chloroplast (cp_Boe) and mitochondrion (mt_Boe) genome sequences of ‘Börner’ from SMRT reads. All bioinformatics tools were applied with default parameters unless otherwise noted.

Genomic DNA was extracted from young leaves of cultivar ‘Börner’ (3) and sequenced on a Sequel I sequencer (1Mv3 SMRT cells, binding kit v3.0, sequencing chemistry v3.0, all from PacBio). Potential plastid or mitochondrial reads were filtered by BLASTN (BLAST 2.7.1+) searches (4) against plastid or mitochondrial sequences (RefSeq release 91). The following criteria were used: read length, above 500 nucleotides (nt); identity, above 70%; query coverage, above 30%. The 292,574 potential plastid reads (2,715,983,671 nt in total; *N*_50_, 12,829 nt) and the 426,918 potential mitochondrial reads (3,928,350,102 nt; *N*_50_, 12,624 nt) were separately assembled with Canu v1.7 (5). Each longest contig displayed high similarity to the chloroplast (6) or mitochondrion (7) genome sequence of *V. vinifera*. Subsequently, Bandage (8) was used to confirm that the assembly was correct. Overlapping end sequences from the circular genomes were manually trimmed, and the start was aligned to that of the grapevine reference sequences. The assemblies were polished three times with Arrow (SMRT Link release 5.1.0.26412). The last round of polishing was carried out with the start shifted to the opposite position of the sequence.

**FIG 1:**
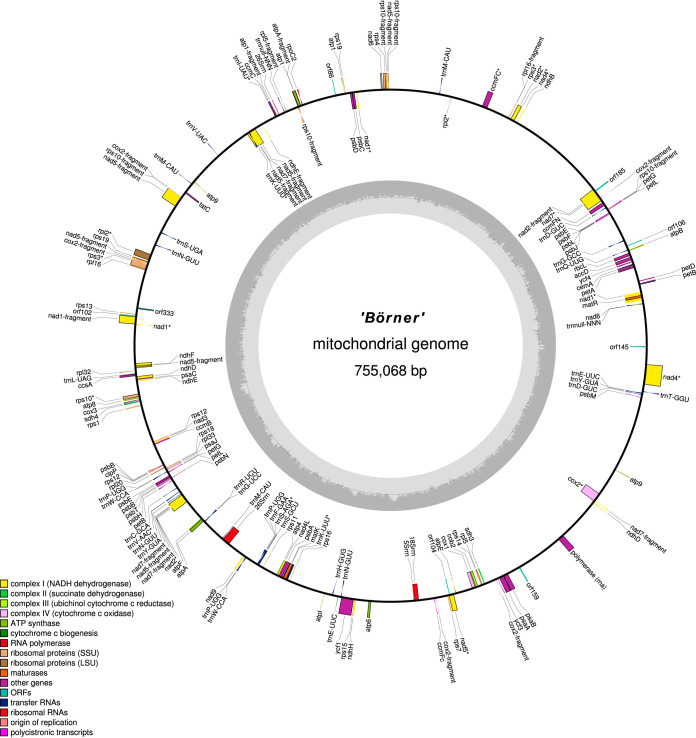
Annotation of the ‘Börner’ mitochondrial genome. The annotation was created with GeSeq and visualized with OGDRAW. Genes containing introns are marked with an asterisk (*).

To aid annotation, RNA was extracted from ‘Börner’ tissues using the peqGOLD plant RNA kit (Peqlab) according to the manufacturer’s instructions. Indexed Illumina sequencing libraries were prepared from 1,000 ng total RNA according to the TruSeq RNA Sample Preparation v2 Guide. The resulting transcriptome sequencing (RNA-Seq) libraries were pooled in equimolar amounts and sequenced in a 2 × 100-nt paired-end format on a HiSeq 1500 instrument.

cp_Boe (161,008 bp; GC content, 37.4%) and mt_Boe (755,068 bp; GC content, 44.3%) were annotated with the Web service GeSeq v1.66 (specific settings for cp_Boe: annotate plastid IR enabled, HMMER profile search [9] enabled, reference sequence *V. vinifera* chloroplast annotation [6], and MPI-MP chloroplast references enabled; specific settings for mt_Boe: reference sequence *V. vinifera* mitochondrion annotation [7]; settings for both: tRNA annotators tRNAscan-SE v2.0 [10, 11], ARAGORN v1.2.38 [12] with “Allow overlaps” and “Fix introns” enabled) (13), which uses OGDRAW v1.3 (14, 15) to visualize the annotation (Fig. 1). RNA editing sites were determined (16) using RNA-Seq data from five different ‘Börner’ tissues. A total of 133 genes with 90 editing sites were identified for cp_Boe, encoding 85 mRNAs, 39 tRNAs, 8 rRNAs, and 1 pseudogene. For mt_Boe, 172 genes with 624 editing sites were identified that encode 67 mRNAs, 38 tRNAs, 4 rRNAs, and 63 pseudogenes/gene fragments. While cp_Boe confirms the maternal inheritance of the chloroplast from *V*. *riparia* due to its high similarity to the chloroplast sequence from *V*. *riparia* voucher Wen 12938 (17), mt_Boe is the first mitochondrion genome sequence from *V*. *riparia* and differs from the *V. vinifera* mitochondrion (7) at 141 positions in the coding regions.

### Data availability.

‘Börner’ RNA-Seq reads (leaves, ENA accession no. ERR3894001; winter leaves, ERR3895010; inflorescences, ERR3894002; tendrils, ERR3894003; roots, ERR3895007), raw SMRT sequence reads (plastid, ERR3610907; mitochondrion, ERR3610837), and chloroplast and mitochondrion genome sequences, including annotation, have been deposited in GenBank/DDBJ/ENA (cp_Boe, ENA accession no. LR738917; mt_Boe, LR738918) under project no. PRJEB34983. The RNA editing tables, coding sequences, and protein sequences of genes subject to RNA editing in edited and unedited form are available as data publications (cpBoe_RNAedit, https://pub.uni-bielefeld.de/record/2941430; mtBoe_RNAedit, https://pub.uni-bielefeld.de/record/2941437).
